# Salt Consumption and Myocardial Infarction: Is Limited Salt Intake Beneficial?

**DOI:** 10.7759/cureus.13072

**Published:** 2021-02-02

**Authors:** Ivan Nikiforov, Charvi Shah, Anish kumar Kanukuntla, Jagan Mohan Rao Vanjarapu, Pratiksha Singh, Satish Tadepalli, Pramil Cheriyath, Vinod Nookala

**Affiliations:** 1 Internal Medicine, Hackensack Meridian Health, Ocean Medical Center, Brick, USA; 2 Internal Medicine, Rutgers University, New Brunswick, USA; 3 Internal Medicine, Mamata Medical College, Khammam, IND; 4 Internal Medicine, University of Pittsburgh Medical Center Pinnacle, Harrisburg, USA; 5 Internal Medicine, Community Medical Center, Toms River, USA

**Keywords:** salt consumption, cardiovascular risk, myocardial infarction, blood pressure, dietary sodium, salt, hypertension

## Abstract

Introduction: Sodium is an essential mineral that plays a crucial role in the maintenance of normal cellular homeostasis, regulation of fluid and electrolytes, and blood pressure (BP). Due to the presence of sodium in a variety of regularly consumed food products, the deficiency of sodium is extremely unlikely. On the other hand, excess intake of dietary sodium is observed in many populations as it is generally used in most food products. Existing guidelines recommend lowering salt consumption for better cardiovascular health; these dietary sodium intake recommendations are not reassuring as the evolving studies show evidence that there is a higher risk of cardiovascular disease (CVD) with low sodium consumption. The aim of this study was to identify the association between salt consumption and myocardial infarction (MI).

Methods: The National Health and Nutrition Examination Survey (NHANES) data between 2017- 2018 was analyzed to examine the association between sodium intake (use in daily meal preparation) and reported history of MI. Logistic regression was used to assess for significant differences between the groups and calculated odds ratios while adjusting for confounders.

Results: A total of 4626 participants were included in the study, with a mean age of 66 ± 11 years in those with a history of MI (n = 212). Amongst these participants, those with salt consumption "Occasionally used" or "Very often used" were less likely to have suffered from MI than those who "Never used" salt in meal preparation. Multivariable logistic regression was performed to control for confounders. “Occasionally used” compared to “Never used” odds ratio was 0.5227 (95% confidence interval (CI); 0.3053-0.9009 p = 0.0184) and “Very often used” compared to “Never used” odds ratio was 0.5033 (95% CI; 0.2892-0.8799 p = 0.0152).

Conclusion: After adjusting for confounders, the participants that used salt more liberally during meal preparation were less likely to have MI than those who minimally or never used salt in meal preparation.

## Introduction

Sodium is an essential extracellular cation that helps maintain water in the extracellular compartment by contributing to the osmotic pressure [1]. Salt in the form of sodium chloride is the primary sodium source, accounting for nearly 95% of daily intake, with the vast majority being excreted by the kidney. In individuals with normal renal function, the kidney can regulate the sodium levels over a wide range of sodium intake without significant changes in the blood pressure (BP). Excess intake of dietary sodium is linked with high BP, and a few studies showed a positive association between increased sodium intake and hypertension [2]. As hypertension is the leading risk factor for cardiovascular disease (CVD) globally, reducing sodium intake has emerged as a proposed target for the reduction of cardiovascular risk [3-5]. Several clinical trials showed evidence of reduced BP by restricting sodium intake [6]. These findings have led to a proposal of decreasing salt consumption, from a current mean intake of nearly 4 g/day to <2 g/day (i.e., more than half current intake) by societies and medical associations [5,7]. The average sodium consumption is between 3-6 g per day in the global population (≈95%) [7,8].

However, there is inconsistent evidence linking sodium intake with the cardiovascular risk that has resulted in considerable controversy about optimal sodium intake for cardiovascular health. Low sodium consumption has a multidimensional effect on the cardiovascular system, including BP, sympathetic activity, insulin sensitivity, and lipid levels [9]. Thus, coming to the conclusion that low sodium consumption reduces adverse cardiovascular events exclusively based on its BP lowering effects may not be accurate.

The mechanisms by which salt intake could affect blood lipids are not clearly understood. The sympathetic nervous system has a significant effect on blood lipid levels, and moderate to severe sodium intake restriction has adverse effects on serum lipids [10-13]. As the serum lipid levels are closely linked with various CVDs, dietary sodium restriction may contribute to an increase in adverse cardiovascular events.

Research showed that there is a significant elevation in serum low-density lipoprotein (LDL) and total cholesterol with short term low intake of sodium (20 mmol/24 h for a period of one week) in normotensive non-obese individuals with the age range between 19-78 years and in healthy men [12,13]. Thus, the amount and the duration of sodium intake can influence the lipid levels in the serum. The aim of this study was to identify if there is an association between salt consumption and a past history of myocardial infarction (MI).

## Materials and methods

Data for 4626 participants recorded through the National Health and Nutrition Examination Survey (NHANES) (2017-2018) was collected and analyzed. Individuals who did not submit the answers to the questions used or were not sure about how to respond to the study's questions were excluded.

NHANES is a cross-sectional survey that is designed to monitor the health and nutritional status of the general non-institutionalized U.S. population, which is selected by a cluster survey design. It is conducted by the Centers for Disease Control and Prevention (CDC) along with the National Center for Health Statistics (NCHS). Included participants were adults aged 18-80 years of age; data regarding age, sex, ethnicity, basal metabolic index, hyperlipidemia, hypertension, MI, smoking status, and salt consumption were collected. 

Salt use was defined by participants' responses to the question "How often is ordinary salt or seasoned salt used in cooking or preparing foods in your household?" The response options were "Never used," "Rarely used," "Occasionally used," and "Very often used." Salt use was treated as an ordinal variable. MI was defined by the response to the question of "Has a doctor or other health professional ever told {you/SP} that {you/s/he} . . .had a heart attack (also called myocardial infarction)?" 

Continuous variables for this study were described as mean and standard deviation. Categorical variables were reported as number and percentage. The Student's t-test was utilized to analyze between-group differences for continuous variables and the chi-square test or Fisher's exact test for categorical variables. A multivariate logistic regression model was used to identify significant predictors for MI. Confounding variables were controlled by being included in the multivariable logistic regression. All analyses were performed with R Studio, version 1.1.456 (RStudio Inc., Boston, MA) at the 0.05 alpha level.

## Results

The study population included 4,626 individuals aged 20 to 80 years. Of those, 212 had a previous MI, and 4414 did not have a previous MI (control group). The MI group's average age was 66.2 ± 11.2 years, while the non-MI control group was 50 ± 17.4 years, p<0.0001. The percentage of females in the MI group was 29.25%, while the control group consisted of 53.01% females, p-value <0.0001. Those who identified as non-white were less prevalent in the MI group than the controls 47.16%, compared to 65.15%, respectively, p<0.0001 (Table 1).

**Table 1:** Demographics of the study population MI - myocardial infarction; SD - standard deviation.

	MI Group		No MI Group		p-value
Age - Mean (SD), range	66.2 (11.2)	22 - 80	50.3 (17.4)	20-80	<0.0001
Gender (Female)	62	29.25%	2340	53.01%	<0.0001
Race (Non-White)	100	47.16%	2876	65.15%	<0.0001

**Figure 1:**
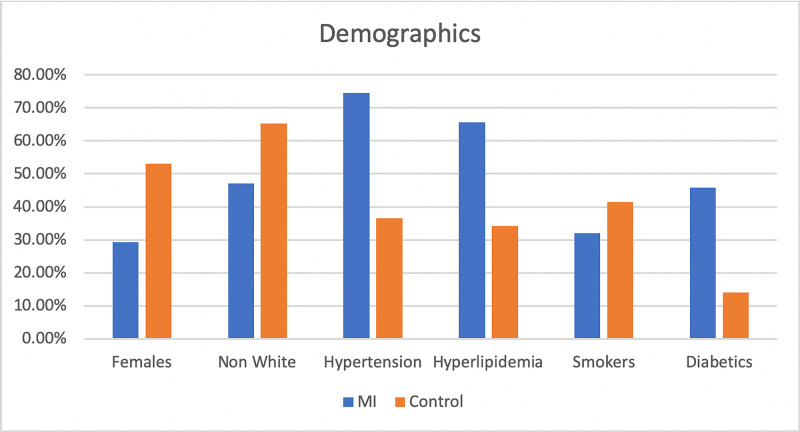
Bar diagram showing the comparison between myocardial infarction (MI) and control groups The figure depicts the differences in race, sex, and various risk factors for MI between the two groups.

Comorbidities were more common in the MI group (Table 2) (Figure 1). Hypertension was roughly twice as likely to be found in the MI group, 74.52% versus 36.52% in the control group, p<0.0001. Hyperlipidemia was also found at a substantially greater rate in the MI group compared to the control group, 65.57% versus 64.21%, p<0.0001 (Table 2). Diabetes was more prevalent in the MI group compared to the control group, 45.75% versus 14.05%, p<0.0001 (Table 2) (Figure 1).

**Table 2:** Risk factors for MI in the study population MI - myocardial infarction; SD - standard deviation; BMI - body mass index.

	MI Group		No MI Group		p-value
BMI - Mean (SD), range	30.5 (6.9)	17.5 - 61.9	29.9 (7.4)	14.8 - 84.4	0.217
Hypertension	158	74.52%	1612	36.52%	<0.0001
Hyperlipidemia	139	65.57%	1510	34.21%	<0.0001
Smokers (Occasional & Every day)	46	31.94%	717	41.42%	0.0088
Diabetes	97	45.75%	620	14.05%	<0.0001

Amongst the participants, those who used salt "Occasionally" or "Very often" had lower odds of having a history of MI, p-value 0.0184 and 0.0152 respectively, when compared to those that "Never" used salt in meal preparation (Table 3). To control the confounders in the study population, multivariate logistic regression was performed. 

**Table 3:** Salt use and predictors of myocardial infarction (MI)

		Confidence Interval		
	Odds Ratio	Lower Limit	Upper Limit	p-value
Salt Use				
Never	reference			
Rarely	0.9088	0.5367	1.5557	0.7240
Occasionally	0.5227	0.3053	0.9009	0.0184
Very Often	0.5033	0.2892	0.8799	0.0152
Gender				
Male	reference			
Female	0.5778	0.3809	0.8599	0.0081
Age	1.0415	1.0259	1.0581	0.0001
Race				
White	reference			
Other Hispanic	0.6809	0.2892	1.4142	0.3366
Mexican Hispanic	0.3506	0.1310	0.7846	0.0196
African American	0.5689	0.3511	0.8997	0.0184
Other	0.7969	0.4343	1.3932	0.4426
Hypertension				
Present	reference			
Absent	0.4879	0.3158	0.7420	0.0010
Hyperlipidemia				
Present	reference			
Absent	0.6813	0.4605	1.0014	0.0524
Smoking				
Non-Smokers	reference			
Some Days	0.7475	0.2515	1.7903	0.5531
Every Day	1.4742	0.9467	2.2774	0.0823
Diabetes				
Present	reference			
Absent	0.4616	0.3136	0.6819	0.0001

## Discussion

According to the CDC, heart disease is the most common cause of death in the United States [14]. Annually, there are roughly 1.5 million MI cases in the United States [15]. While many modifiable risk factors have been associated with MI, such as diabetes, hypertension, hyperlipidemia, and obesity, dietary or daily salt use is not among them [15]. While higher sodium intake has been shown to lead to hypertension, which is one of the primary risk factors for MI, current studies only focus on BP reduction and MI prevention [16]. It is unclear if the salt restriction is protective against MI if a BP goal is reached. This study's findings seem to indicate that salt restriction may be harmful in patients with controlled BP.

This study looked at the association between the amount of salt consumption in meal preparation and MI. Of the total 4,626 participants, 212 had a history of MI. Those that used salt "Occasionally" or "Very often" had lower odds of having a history of MI, p-value 0.0184 and 0.0152 respectively, when compared to those that "Never" used salt in meal preparation (Table 3). As established in many other studies, women had a lower chance of having MI than men, p-value 0.0081 [17,18]. Additionally, Mexican Hispanics and African Americans had lower odds of having an MI than Whites, p-value 0.0196, and 0.0184, respectively (Table 3).

Several previous studies have examined salt intake and cardiovascular events and mortality, and all-cause mortality that have had similar findings. Stolarz-Skrzypek et al. found that lower sodium excretion was associated with a higher risk of CVD mortality [19]. Kalogeropoulos et al. found that salt intake under 1500 mg/day was associated with higher mortality compared to a salt intake of 1500-2300 mg/day. Salt intake above 2300 mg/day resulted in non-statistically significant rates of higher mortality [20]. Meller et al. repeated the same study in adults aged 70 to 79 years of age and found that the group that consumed 1500 mg-2300 mg/day had the lowest CVD rates, heart failure, and mortality [21]. However, once the results were adjusted for confounding variables, there were no statistically significant differences in any outcomes between the three groups. O'Donnell et al. found that urinary sodium excretion of less than 3 g/day had the highest odds of dying or having a CVD event [22]. A meta-analysis carried out by Strazzullo et al. showed lower mortality for the group that had 2.7-5 g/day of sodium had lower mortality than the group that consumed less than 2.7 g/day of sodium [23].

On the other hand, several studies have shown an increased risk of cardiac events and mortality with high sodium intake. O'Donnell et al.'s previously mentioned study had the second-highest adverse outcomes in the group with>7 g/day of sodium excretion [22]. Cook et al. found that as sodium consumption increased, all-cause mortality increased, although none of the results were significant [24]. A meta-analysis carried out by Aburto et al. showed a higher risk of cardiovascular mortality, but not all-cause mortality [6]. Finally, when Strazzullo et al. used the same data set as Aburto et al. and only compared moderate sodium intake 2.7 g/day and high sodium intake >5 g/day, there was an increase in cardiovascular mortality and all-cause mortality [23]. While some studies show increased mortality in groups that consume low amounts of sodium and others do not show any benefit to a low sodium diet, ultimately, there have been no studies that show that low sodium consumption reduces mortality in the general population. Studies that clearly show increased mortality with high sodium intake tend to focus on relatively higher sodium consumption, only showing substantial results with a reduction of sodium intake 4.8 g/day or greater [25]. Unfortunately, the average sodium consumption for adults in all countries outside of Asia is already under 5 g/day [7].

This study has numerous strengths. It looks at a large and diverse population for the association between daily salt consumption and MI. Unlike other studies, it does not look at specific amounts of salt used but at more general self-reported salt use in meal preparation. This is substantially more practical and applicable to the general population, as it is improbable that most people take the time to measure how much salt they use daily stringently. The data comes from a reliable source, NHANES, widely used in research and trend analysis in the United States.

This study has a few limitations. An important factor to consider while interpreting the data of this study is survivor bias. Only the individuals that survived MI were included in this study. This is especially a problem since African Americans were less likely to survive MI than Whites, and in the data, however, it was shown that African Americans were less likely to have a history of MI than Whites [6]. As the data regarding salt consumption was treated as an ordinal variable, it is practically not possible to quantify the intake of salt in the meal preparations of the study population. Also, a significant proportion of sodium content in an individual's diet comes from consuming sodium that has been pre-added to spices, sauces, canned food, processed meats, snacks, etc. Usually, patients do not take those sources of sodium into consideration while reporting the amount of sodium intake in questionnaires. Ultimately, this study shows that in the general population, salt use reduction in meal preparation is associated with higher odds of having MI. It adjusts for numerous factors such as race, gender, age, hypertension, and hyperlipidemia. Further studies that focus on the quantity of daily salt consumption are needed to support the observed findings.

## Conclusions

Our study showed that those who use salt more liberally while preparing meals are less likely to have a history of MI. Overall, the average amount of salt consumed per day by the general public in the United States would be considered moderate, according to most of the studies. Over the last decade, an emerging number of studies have found the association between increased mortality and low sodium intake. Generally, moderate salt intake seems to have the best outcomes in terms of mortality and morbidity compared to high or low salt intakes. High salt intake has been established to cause hypertension, which seems to be the primary factor in adverse outcomes. More research is needed in this area to evaluate this association further.
